# Analysis of the Protein Phosphotome of *Entamoeba histolytica* Reveals an Intricate Phosphorylation Network

**DOI:** 10.1371/journal.pone.0078714

**Published:** 2013-11-13

**Authors:** Tamanna Anwar, Samudrala Gourinath

**Affiliations:** School of Life Sciences, Jawaharlal Nehru University, New Delhi, India; University of Hyderabad, India

## Abstract

Phosphorylation is the most common mechanism for the propagation of intracellular signals. Protein phosphatases and protein kinases play a dynamic antagonistic role in protein phosphorylation. Protein phosphatases make up a significant fraction of eukaryotic proteome. In this article, we report the identification and analysis of protein phosphatases in the intracellular parasite *Entamoeba histolytica*. Based on an *in silico* analysis, we classified 250 non-redundant protein phosphatases in *E. histolytica.* The phosphotome of *E. histolytica* is 3.1% of its proteome and 1.3 times of the human phosphotome. In this extensive study, we identified 42 new putative phosphatases (39 hypothetical proteins and 3 pseudophosphatases). The presence of pseudophosphatases may have an important role in virulence of *E. histolytica.* A comprehensive phosphotome analysis of *E. histolytica* shows spectacular low similarity to human phosphatases, making them potent candidates for drug target.

## Introduction

The eukaryotic protozoan parasite *Entamoeba histolytica* is the causative agent of amoebiasis, a global health threat responsible for an estimated 40–50 million cases of invasive colitis or liver abscess and up to 100,000 deaths per year [1], [2]. Although the parasite has a worldwide distribution, it predominantly affects individuals of lower socioeconomic status, who live in developing countries [2]. Protein phosphorylation is a key post-translational modification that is regulated by the competing activities of protein kinases (PK) and protein phosphatases (PP) [3]. The net phosphorylation state relies on a delicate balance between PKs, which catalyse phosphate addition, and PPs, catalysing phosphate removal. Thus it is not surprising that disease conditions often correlate with alteration of the cell phosphorylation profile as a consequence of a perturbation of kinase and/or phosphatase activities [4]–[6].

PKs are currently the pharmaceutical industry’s second largest drug targets, which are extensively studied [7]. In contrast, the role of phosphatases in disease has only recently come to research forefront. The extent of phosphorylation at a particular site can be regulated by changing the activity of the cognate PK or PP or both. [8]. About 30% of all proteins can be regulated by phosphorylation [8], [9]. Many cellular signalling events are regulated by phosphorylation and de-phosphorylation mediated by the opposing actions of protein kinases and phosphatases. Similar to kinases, protein phosphatases are emerging as drug targets, but poor cell permeability of inhibitors has limited the development of drugs targeting these enzymes. Recent advances in the understanding of the role of phosphatases in the pathogenesis of *E. histolytica* have opened up an exciting avenue for drug development, where protein phosphatases can act as drug targets.

Anamika *et al.,* 2007 identified 307 PKs in *E. histolytica* [10] which is less than half the size of the human kinome consisting of 507 putative PKs, differing in numerous ways from kinases in the mammalian host [3]. *E. histolytica* is reported to have greater than 100 PPs, which dephosphorylate proteins [11]. Since the phosphorylation status of any protein is controlled by both kinases and phosphatases, the latter can be exploited as therapeutic targets as well [4], [12]–[13].

Here we present detailed analysis of PPs in *E. histolytica.* Through *in silico* analysis of protein sequences and structural domains we identified 250 PPs in *E. Histolytica,* which are more than PPs identified in human genome. Phosphoprotein phosphatases (PPP) form the largest family of PPs. Many unusual phosphatases involved in protein phosphorylation make *E. histolytica* different from other eukaryotic organisms. Structural analysis reveals that *E. histolytica* PPs show low similarity to human PPs, making it good for drug targeting.

## Materials and Methods

The complete set of predicted protein sequences from the ORFs of the *E. histolytica* genome has been obtained from NCBI (version 2010) [14]. We have surveyed the genome, for PPs using sensitive sequence analysis methods as described below: Domain assignments have been made for PP catalytic domain containing gene products using: (1) HMMer by querying each of the phosphatase domain containing proteins against all the protein family HMMs available in the Pfam database release Pfam 26.0 (November 2011, 13672 families) [15] and (2) InterProScan5 by querying each of the phosphatase domain containing proteins against the 16409 protein families and 6850 domains available in the InterPro database (InterPro 41.0 13th February 2013) [16]. InterPro, provides a powerful tool for protein sequence classification and function prediction. InterPro integrates all the protein signature databases into one, list of InterPro domains related to PPs is given in Table S1 in file SI. It has been used in many genome annotation projects, as well as by UniProt curators for individual protein sequence annotation [17]. We have chosen protein sequences with phosphatase domain having e-value score of 10^−5^. InterPro picked all the sequences predicted by Pfam as it integrates Pfam in its search. CD-HIT program [18] was used to eliminate redundant sequences which are indicated by 100% sequence identity. CELLO v.2.5 was used for subcellular localization prediction.

Structural domain analysis was carried out using Phyre2 (Protein Homology/AnalogY Recognition Engine) that is a web-based service for protein structure prediction [19]. Multiple alignments were constructed using ClustalW [20]. Evolutionary relationships were studied using phylogenetic analysis package Mega4.0 [21]. List of all the tools used in the analysis of PPs is cited in Table S2 in file SI.

## Results and Discussion

**Figure 1:**
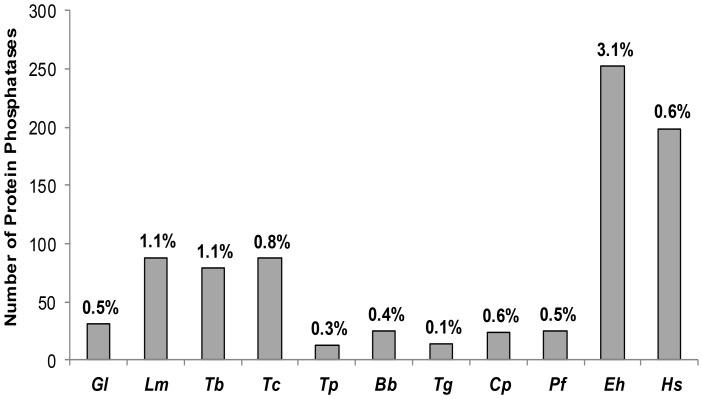
Bar diagram-representing comparison of PPs in the genomes of parasitic protozoa and human. The percentage of protein phosphatases genes in the proteome complement is provided against every bar. Species abbreviations used in the diagram are as follows: *Gl*, *Giardia lamblia*; *Lm*, *Leishmania major*; *Tb*, *Trypanosoma brucei*; *Tc*, *Trypanosoma cruzi*; *Tp, Theileria parva*; *Bb*, *Babesia bovis*; *Tg*, *Toxoplasma gondii*; *Cp*, *Cryptosporidium parvum*; *Pf*, *Plasmodium falciparum*; *Eh*, *Entamoeba histolytica*; *Hs*, *Homo sapiens*.

Protein phosphatases, like many other signaling molecules, can be inhibited or activated by small molecules that occur naturally in the cell [22]. Total number of functional PPs encoded in the genomes of some of the protozoan parasites [23]–[24] is shown in Figure 1. The genome of *E. histolytica* encodes 250 putative PPs which is around ten times the number of PPs encoded in the genome of the malaria parasite *Plasmodium falciparum* [25] and 1.3 times the number of phosphatases encoded in the human genome [26]. It was observed that *E. histolytica* phosphatases differs greatly from human phosphatases, none of the *E. histolytica* PPs has shown >40% similarity to the human PPs.

### Sequence and Structure Domain Analysis

To confirm the presence of phosphatase family domain the 250 PPs retrieved through InterPro were searched for structural phosphatase domains. Phyre2.0 server analysis showed that phosphatase structural domains were present in all the putative PPs (Table S3 in file SI) except for three proteins EAL49728.2, EAL48868.1 and EAL49020.1, but through InterPro it was found that EAL49728.2 had dual specificity phosphatase (DSP) domain, EAL48868.1 had protein tyrosine phosphatase (PTP) -like domain and EAL49020.1 had PPP (PP2A) family domain.

### Distribution of PPs in *E. histolytica*

**Figure 2:**
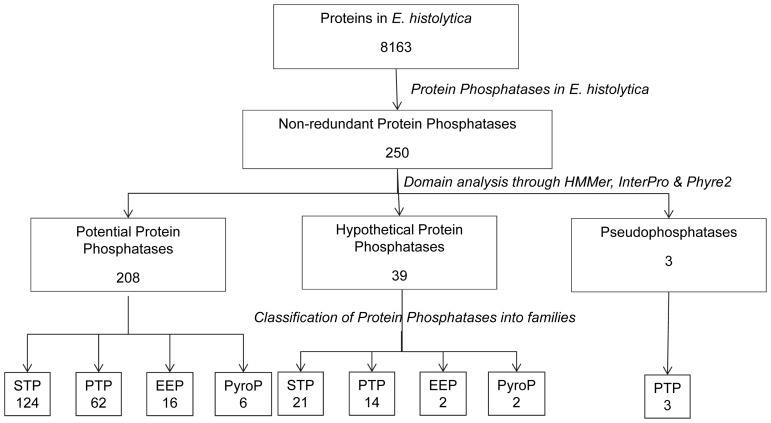
Flow scheme for the assignment of *E. histolytica* PPs obtained at different steps of the analysis.

The *E. histolytica* phosphotome, differs in numerous ways from phosphatases in the mammalian host. The numbers of PPs obtained at different steps of the analysis are shown in Figure 2. The distribution of PPs into various families is summarized in Table 1 along with subfamily assignments and other domains that are tethered to phosphatase catalytic domains. Among the 250 putative PPs in the dataset, 145 are likely to be Protein Ser/Thr phosphatases (STPs), 79 PTPs, 18 endonuclease/exonuclease/phosphatase (EEP) and 8 pyrophosphatases (Table 2). PPs are classified based on characteristics such as sequence, structure and phosphoamino-acid specificity. According to Szoor, 2010 protein phosphatases are classified into four major groups based on catalytic signature motifs and substrate preferences: phospho-protein phosphatase (PPP), metallo-dependent protein phosphatase (PPM), aspartate-based phosphatases (FCP) (the members of these three groups are ser/thr specific phosphatases) and the distinct group of (PTP). Haloacid dehalogenase (HAD) is considered as a member of FCP family [27] and protein histidine phosphatases (PHP) are a sub-group of PTP superfamily [28].

**Table 1:** Classification of protein phosphatases into families.

Phosphatase families	Examples of members
*Protein serine/threonine phosphatases*	
PPP family	PP1, PP2A, calcineurin, PP5
PPM family	PP2C
FCP family	FCP, HAD family (Asp-based)
*Protein tyrosine phosphatases*	
Class I Cys-based PTPs	Classical PTPs, DSPs
Class II Cys-based PTPs	CDC25A, CDC25B, CDC25C
Class III Cys-based PTPs	LMPTP
Histidine Phosphatases	Histidine-dependent acid phosphatases
*Exonuclease-Endonuclease-Phosphatase*	Endonucleases, Inositol polyphosphate 5-phosphatases (INPP5), Synaptojenin proteins
*Pyrophosphatases*	DeoxyutpPyrophosphatase (dUTPase), Inorganic Pyrophosphatase

This classification was compiled from Ref. 22, 31, 40.

**Table 2:** Distribution and sub-cellular localization of PPs in *E. histolytica.*

Phosphatases	Sub-cellular Localization	Class	No.	Total No.
Serine/Threonine Phosphatases	Nuclear, Plasma Membrane,Cytoplasmic, Extracellular,Mitochondrial	PPP	64	145
		PPM	42	
		FCP (HAD Family)	39	
PTPs	Nuclear, Plasma Membrane,Cytoplasmic, Chloroplast	PTPc	22	79
		DSPc	22	
		CDC25	14	
		LMWPTPASE	2	
		Histidine Phosphatase	19	
Pyrophosphatase	Nuclear, Cytoplasmic,Chloroplast	Deoxyutp Pyrophosphatase Family	7	8
		Inorganic Pyrophosphatase Family	1	
Endonuclease/Exonuclease/Phosphatase	Cytoplasmic		18	18
**Total**				**250**

Members of the EEP superfamily belong to different families of enzymes, like endonucleases, inositol 5-phosphatases (INPP5) are Mg^2+^ dependent and Inositol 4-phosphatases belongs to PTP family [29]–[31], while pyrophosphatases it has not been included in any of the classifications available (Table 1).

### Protein Ser/Thr Phosphatases (STPs)

Protein STPs are encoded by three unrelated gene families, PPP, PPM and FCP [32]. We predicted a total of 145 STPs in *E. histolytica*. PPP family include protein phosphatase1 (PP1), PP2A, PP2B, PP4, PP5, PP6, and PP7. Out of 145 putative STPs in *E. histolytica*, 64 PPs belong to PPP family. Protein phosphatases of the PPM (represented by PP2C) family are present in both eukaryotes and prokaryotes. The PPM family includes PPs dependent on manganese/magnesium ions (Mn^2+^/Mg^2+^). In contrast to PPP, members of the PPM family do not have regulatory subunits but contain instead additional domains and conserved sequence motifs that may help determine substrate specificity. In *E. histolytica,* 42 PPM family members were identified. The FCP family which dephosphyralates the carboxy terminal of RNA Polymerase II was most recently recognised, these are widely distributed among eukaryotes [33]. In our analysis we found 39 PPs belonging to FCP (HAD-like) family (Table 2), following the recent classification of Szoor, 2010 we have placed HADs as member of FCP family. The HAD family, includes phosphoesterases, ATPases, phosphonatases, dehalogenases, and sugar phosphomutases acting on a remarkably diverse set of substrates [34], [35]. Kutuzov *et. al.,* 2008 have reported 114 STPs in *E. histolytica* [23], while in the present analysis a total of 145 STPs were identified. PPPs form the largest family among STPs superfamily.

### Protein Tyrosine Phosphatases (PTPs)

PTPs are the key regulatory components in signal transduction pathways, cell cycle control and are important in the control of cell growth, proliferation, differentiation and transformation. PTPs belong to three evolutionarily unrelated classes: Class I, Class II and Class III cys-based PTPs. Class I cys-based PTPs consist of classical PTPs and DSPs. Among the 79 PTPs identified in *E. histolytica* 22 are putative classical PTPs and 22 belonged to DSPs. Classical PTPs are strictly tyr-specific sharing a common cysteine-based mechanism of catalysis [9], [36]. DSPs dephosphorylate different combinations of tyr and ser/thr, as well as non-protein substrates. These enzymes have low sequence similarity beyond the cysteine-containing motif and smaller catalytic domains than the classical PTPs. The class II cys-based PTPs comprise a small group of cell cycle regulators (CDC25) phosphatases, *E. histolytica* has 14 such PPs. Their catalytic machinery is very similar to that of class I enzymes, they are structurally unrelated [37]. The class III cys-based protein phosphatases include low molecular weight PTPs (LMPTP), the parasite has 2 such phosphatases. LMPTPs are highly conserved throughout evolution from yeasts to man and highly homologous genes are even seen in prokaryotes. [38]. The protein histidine phosphatases (PHP) is a large functionally diverse group of proteins. Among the 79 PTPs identified 19 were PHPs. In contrast to cysteine-dependent PTPs, PHPs utilize histidine, rather than cysteine, for substrate dephosphorylation [39].

### Exonuclease-Endonuclease-Phosphatases (EEPs)

EEP family is a structural domain found in the large family of proteins. EEPs include magnesium dependent endonucleases and many phosphatases involved in intracellular signalling [40], [41]. This large superfamily includes a diverse set of proteins (Table 1). In *E. histolytica* 18 PPs belonging to EEP family were predicted, 8 phosphatases from classical EEPs and 10 belonging to inositol polyphosphate sub-group.

Inositol phosphatases belong to different families of enzymes; PTEN (phosphotase and tensin homolog) and myotubularin inositol 3-phosphatases belong to PTP superfamily. Inositol 4-phosphatases share with PTPs the conserved active site signature CX-5-R (P-loop motif). INPP5 are Mg^2+^-dependent enzymes related to endonucleases [29]–[31], showing distinct sequence and biochemical characteristics to classic eukaryotic lipid phosphatases having no homologues in humans [29]. In *E. histolytica* we identified 6 INPP5 family members with sequence ids EAL44576.2, EAL45154.1, EAL44267.1, EAL44027.1, EAL50984.1 and EAL45706.2.

### Pyrophosphatases

In parasites, a proper ion balance is essential for them to be able to invade and live in other organisms. Membrane-bound pyrophosphatases cannot be found in humans but they are crucial for the survival of protozoan parasites. Membrane proteins in general are important targets for drugs [42]. In *E. histolytica* we observed 7 pyrophosphatases from Deoxyutp Pyrophosphatase (dUTP) family and 1 from Inorganic Pyrophosphatase family (Table S3 in file SI). dUTPases helps in preventing the concentration of dUTP rising above a base level in the cell. Inorganic pyrophosphate has been shown to be necessary for the growth of *Escherichia coli* and for yeast mitochondrial function [43], [44]. Understanding the structure and function of pyrophosphatases will help us in designing specific drugs to disturb its function.

### Unusual Domain Combinations

**Figure 3:**
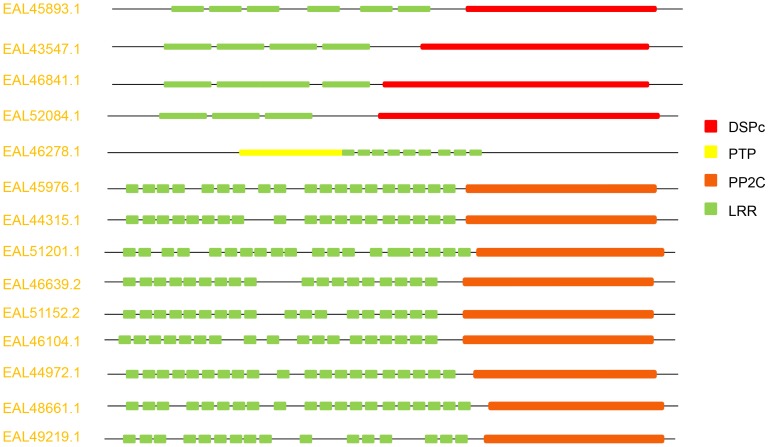
Protein phosphatase genes in *E. histolytica* with varying number of LRR domains.

The presence of varying numbers of leucine-rich repeat (LRR) domain is an unusual feature of some of the phosphatases, which may be involved in protein–protein interactions. A list of various PPs with LRRs identified in *E. histolytica* genome is presented in Figure 3. It consists of five phosphatases having PTP family domain (four DSPs and one classical PTP) and 9 STPs belonging to PPM family. The LRRs may be associated in interaction with host cell and pathogenesis of the parasite. Unexpectedly, LRRs have been shown to be associated with microbial virulence factors helping in the interaction with host cells and infection establishment [45].

### Pseudophosphatases

We have observed several proteins with non-functional phosphatase domain, these proteins were named as pseudophosphatases. Several members of the PTP superfamily possess conserved domains with core features of a PTP, but which lack residues that are critical for catalysis [46]. Pseudophosphatases are most prevalent among the Myotubularins (sub-family from PTP superfamily). Three such proteins were predicted in *E. histolytica* genome, out of which two were having a PK domain, a PP domain (PTPs Family) and LRRs, the other one had a PK domain, a PP domain but no LLRs (Table 3). In other protozoans such as *Giardia lamblia, Tetrahymena thermophila,* and *Dictyostelium discoideum* also similar domain architecture (kinase+phosphatase) is seen, indicating evolutionary conservation of these proteins [47]. Recently, it has been demonstrated that inactive myotubularins form complexes with the active enzymes. These interactions regulate both the enzymatic activity and the subcellular location of the active phosphatase [6]. LRR-DSPs and kinatases (a DSP domain with two pseudokinase domains and LRRs) in bacteria show similarity to LRR proteins, suggesting that these are involved in virulence in this parasitic protozoan also [47]. It appears that the presence of such PPs may have an important role in virulence.

**Table 3:** Putative phosphatases with multiple domains in *E. histolytica*.

Acc. No.	Kinase	Phosphatase	LRR
EAL46608.2	Yes	Yes	Yes
EAL47388.1	Yes	Yes	Yes
EAL49131.2	Yes	Yes	No

### Unidentified Proteins

There are several hypothetical proteins, which are not studied at all so far but we have identified phosphatase domains in these proteins and we named it as unidentified PPs. Our analysis has added to the list of PPs whose functions are not yet well understood. We have identified at least 39 gene products with a clear similarity to PPs, but to the best of our knowledge these genes are unexplored by experimental analysis. A list of these phosphatases with their domain assignments is shown in Table S4 in file SI. The catalytic domains in most of these hypothetical proteins could be associated to known families of PPs because of a high similarity of the catalytic regions.

### Phylogenetic Classification

**Figure 4:**
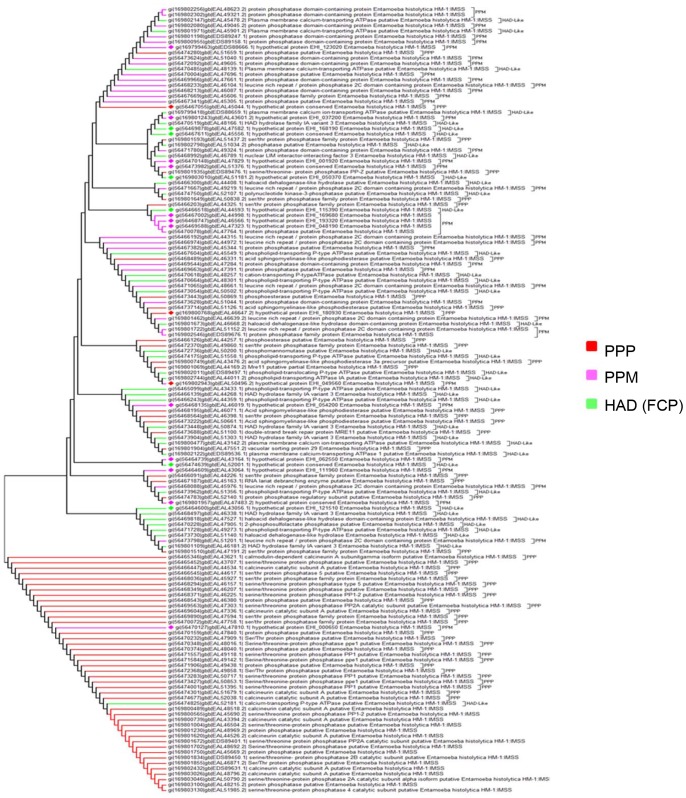
Phylogenetic tree representing relationship among STPs. Branches with diamond shapes represents hypothetical proteins.

**Figure 5:**
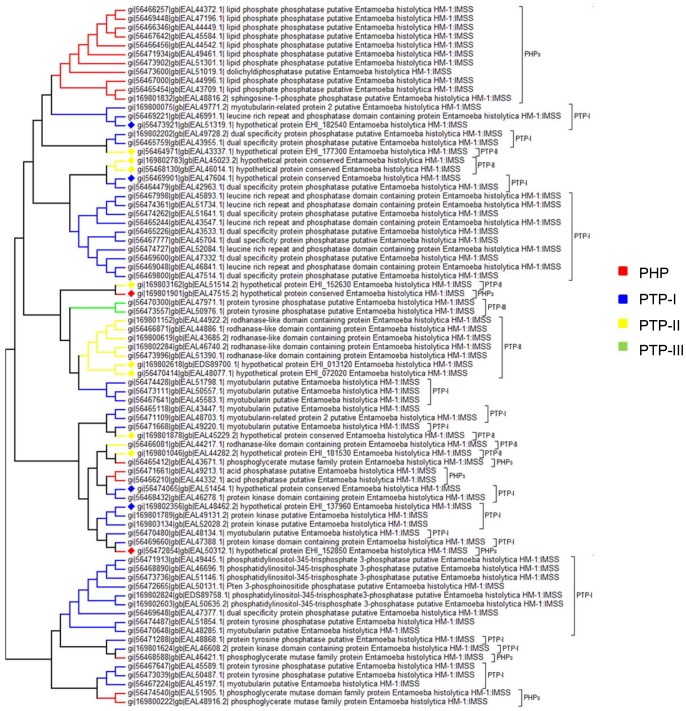
Phylogenetic tree representing relationship among PTPs. Branches with diamond shapes represents hypothetical proteins.

**Figure 6:**
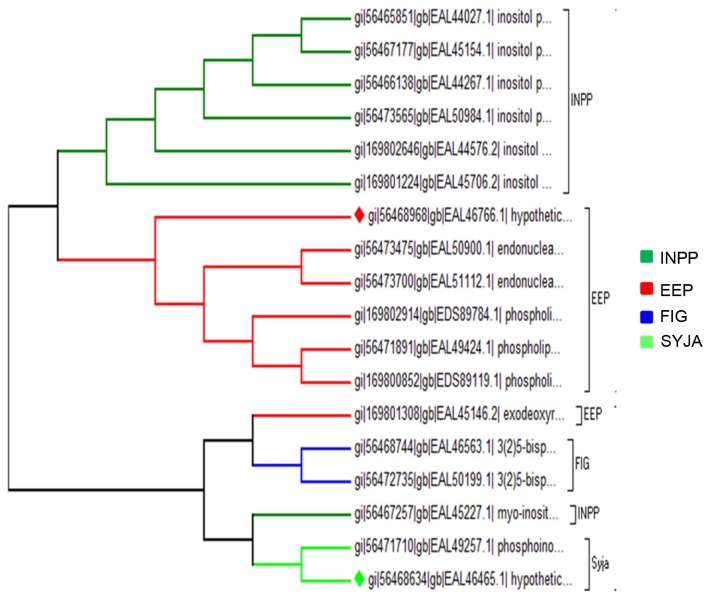
Phylogenetic tree representing relationship among EEPs. Branches with diamond shapes represent hypothetical proteins.

**Figure 7:**
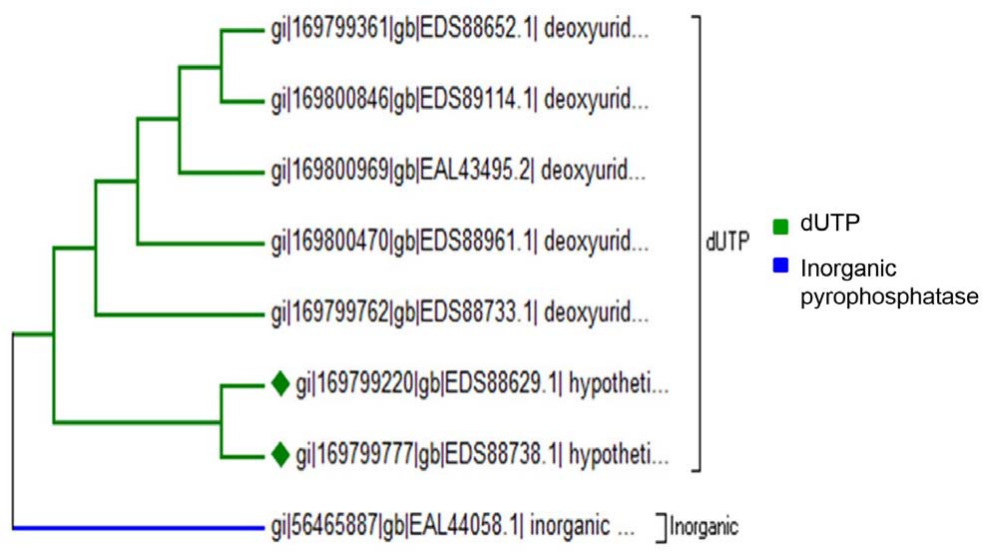
Phylogenetic tree representing relationship among Pyrophosphatases. Branches with diamond shapes represent hypothetical proteins.

Based on the amino acid sequence, two hundred fifty *E. histolytica* PPs could be placed in one of the known families. From the phylogenetic analysis of STP family it was observed that it consists of two branches with one branch consisting strictly of PPPs except for one PPM family member. The other branch composed of sub-branches from all the three families of STP family (Figure 4). PTP family tree shows that PHPs are closely related to PTP-I family than to PTP-II and PTP-III family (Figure 5). Phylogenetic relationship among EEPs shows that inositol polyphosphates are close relatives of classical EEPs, while synaptojenin and FIG family members are distantly related to EEPs and INPPs (Figure 6). Pyrophosphatases form two distantly related families consisting of one branch only of dUTPases and other inorganic phosphatases (Figure 7). The phylogenetic classification also confirms that hypothetical proteins are showing close relationship to the members of the assigned families.

## Conclusions

The dephosphorylation of proteins is catalyzed by PPs, acting antagonistically to PKs. PPs are apparently less attractive drug targets, because they typically act on a broader range of proteins than kinases do. In many parasitic diseases, a gain of specific phosphatase function may contribute to the pathology. In parasites like *E. histolytica* PPs play essential role in signalling mechanism. In such instances, these phosphatases may serve as appropriate drug targets. A comphrensive analysis shows that *E. histolytica* phosphotome is about 3.1% of the total proteome size which is a little more than most of other eukaryotes. A large number of PPs manifests protein phosphorylation as the key mechanism of signal transduction in the parasite. The phosphotome is classified into classical groups STP and PTP, in which STP has highest members with 145 proteins, indicating STPs as the key players in the regulation of the parasite. Several members were seen from the new family of phosphatases (INPP5 family) that do not have homologs in humans. Proteins from the pyrophosphatase family which are crucial for the survival of the parasite were also identified. This indicates that members of the INPP5 and pyrophosphatase family can act as good drug targets. Several phosphatases in combination with LRRs were also seen, the involvement of LRRs in microbial pathogenesis and their capability to bind to a vast array of structurally unrelated ligands make them a potential target for vaccines and new drugs. Few pseudophosphatases were also identified, these are suggested to be involved in virulence of the parasite [47]. In particular, protein phosphorylation is a major currency of signal transduction pathways. Exploring the conditions under which the cells employ two different kinetic mechanisms for dephosphorylation will help us to understand more about the evolutionary adaptation of organisms.

## Supporting Information

File S1 **Table S1–S4.** *Table S1.* List of InterPro domains associated with protein phosphatases. *Table S2.* List of tools used in classification of PPs. *Table S3.* Structural domain analysis of PPs in *Entamoeba histolytica*. *Table S4.* Classification of hypothetical proteins on the basis of InterPro domains with significant e-values.
(DOC)
