# Supplementary material for: Analysis of the Protein Phosphotome of Entamoeba histolytica Reveals an Intricate Phosphorylation Network
Source: PLoS One. 2013 Nov 13;8(11):e78714. doi: 10.1371/journal.pone.0078714 (PMC3827238; doi:10.1371/journal.pone.0078714)
Supplement: File S1 — Table S1–S4. Table S1. List of InterPro domains associated with protein phosphatases. Table S2. List of tools used in classification of PPs. Table S3. Structural domain analysis of PPs in Entamoeba histolytica. Table S4. Classification of hypothetical proteins on the basis of InterPro domains with significant e-values. (DOC) [file pone.0078714.s001.doc]

**Table S1.** List of InterPro domains associated with protein phosphatases.

| InterPro Phosphatases | Families | Domains |
| --- | --- | --- |
| Protein-tyrosine phosphatase | IPR019750, ***IPR007482***, IPR020428, IPR008356, IPR017867, IPR002115,  IPR012265, IPR012266, IPR012153, IPR014392, IPR012151, IPR012152,  IPR016334, IPR004861, ***IPR000106***, IPR016336, IPR000751 | IPR006545, ***IPR017906***, IPR024739, IPR003595, ***IPR000242, IPR001763***,  ***IPR025163, IPR014020*** |
| Dual specificity phosphatase | IPR020417, IPR020405, IPR020420 IPR024950, IPR026067, ***IPR026893*** | IPR025138, ***IPR000340***, ***IPR020422***, ***IPR000387*** |
| Histidine phosphatasesuperfamily | IPR016274, ***IPR000560***, IPR010140, IPR006543, IPR001011, IPR010028,  IPR024927, IPR005519 | ***IPR013078,*** IPR003141, ***IPR016118*,** IPR008963, IPR015914 |
| Serine/threonine protein phosphatase | ***IPR000326***, IPR011230, IPR011152, IPR011240, IPR012365, IPR024173,  IPR011401, IPR014485, IPR014578, IPR014576, IPR015655, IPR012008,  ***IPR011236***, IPR024201 | IPR007312, ***IPR004843***, IPR007312, IPR023190, IPR014787, ***IPR023485***,  ***IPR001932***, ***IPR006186*** |
| HAD-superfamily hydrolase | IPR006386, IPR006381, IPR010021, IPR010025, IPR005833, ***IPR000150,***  ***IPR013954***, IPR010023, IPR010039 | IPR011947, IPR010037, IPR004182, ***IPR023214****,*IPR010033 |
| EEP | IPR022337 | ***IPR005135, IPR000300*** |
| Pyrophosphatases | ***IPR022934***, IPR003763, IPR015993,  IPR003565, IPR004131, ***IPR023537*** | IPR004097 |
| Alkaline phosphatase | IPR001952, IPR026263 | IPR017849, IPR017850 |
| Others* | IPR015768, IPR020691, IPR004464, IPR012765, IPR011310, IPR008343, IPR002786, IPR011158, IPR016965, IPR020243, IPR025899, IPR006551, IPR006550, IPR023733, IPR002828, IPR014577 | IPR025138, IPR014876, IPR011948, IPR023215, IPR026533, IPR003501,  IPR013679, IPR012822, IPR003337 |

*Others include Activin type II/Transforming growth factor-beta II receptor, Fructose-1,6-bisphosphatase, Lipopolysaccharide core heptose(II)-phosphate phosphatase, etc.

InterPro domains identified in *E. histolytica* are represented in bold and italics.

**Table S2.** List of tools used in classification of PPs.

| **Tools** | **Used for Analysis** |
| --- | --- |
| HMMer | Protein domain assignment |
| InterProScan5 | Protein domain assignment |
| CD-HIT | Eliminating redundant sequences |
| Phyre2 | Protein structural domain assignment |
| ClustalW | Multiple alignments |
| Mega4.0 | Phylogenetic analysis package |
| CELLO v.2.5 | Subcellular localization predictor |

***Table S3.*** *Structural domain analysis of PPs in* Entamoeba histolytica

| **Family** | **InterPro Domain** | **Template** | **3d Model** | **Source** | **Members** |
| --- | --- | --- | --- | --- | --- |
| PPP Family | PP2Ac | 1auiA | Serine/Threonine Phosphatase 2b | *Homo sapiens* | 20 |
|  |  | 1jk7a | Metallo-Dependent Phosphatases | *Homo sapiens* | 1 |
|  |  | 1wao4 | Metallo-Dependent Phosphatases | *Homo sapiens* | 1 |
|  |  | 2jogA | Calmodulin-Dependent Calcineurin A | *Homo sapiens* | 1 |
|  |  | 2p6ba1 | Metallo-Dependent Phosphatases | *Homo sapiens* | 1 |
|  |  | 3dw8B | Serine/Threonine-Protein Phosphatase 2a | *Homo sapiens* | 1 |
|  |  | 3icfB | Serine/Threonine-Protein Phosphatase | *Saccharomyces cerevisiae* | 1 |
|  |  | 2nxfa1 | Metallo-Dependent Phosphatases | *Danio rerio* | 7 |
|  |  | 2yvta1 | Metallo-Dependent Phosphatases | *Aquifex aeolicus VF5* | 1 |
|  |  | 3c5wc1 | Metallo-Dependent Phosphatases | *Homo sapiens* | 2 |
|  |  | 1ii7a | Metallophosphoesterase Mpped7 | *Pyrococcus furiosus* | 6 |
|  |  | 1s70a | Metallo-Dependent Phosphatases | *Homo sapiens* | 12 |
|  |  | 1s95a | Metallo-Dependent Phosphatases | *Homo sapiens* | 1 |
|  |  | 1xm7a | Metallo-Dependent Phosphatases | *Aquifex aeolicus VF5* | 1 |
|  |  | 1z2wa1 | Metallo-Dependent Phosphatases | *Mus musculus* | 1 |
|  |  | 3d03a1 | Metallo-Dependent Phosphatases | *Enterobacter aerogenes* | 1 |
|  |  | 3rl4A | Metallophosphoesterase Mpped5 | *Rattus norvegicus* | 6 |
| PPM Family | PP2C | 1a6qA | Phosphatase 2c | *Homo sapiens* | 29 |
|  | PP2Cc superfamily | 1txoa | PP2C-Like | *Mycobacterium tuberculosis* | 12 |
| FCP Family (HAD-like) | 2-ph_phosp superfamily | 2yyvB | Probable 2-Phosphosulfolactate Phosphatase | *Thermotoga maritima MSB8* | 1 |
|  | HAD-superfamily hydrolase | 1yj5a | 5' Polynucleotide Kinase-3' Phosphatase Catalytic Domain | *Mus musculus* | 1 |
|  | HAD_like superfamily | 2fdra | Hydrolases/Phosphatases-Like Fold Protein | *Agrobacterium tumefaciens str. C58* | 1 |
|  | HAD_like | 1nf2a | HAD-Like | *Thermotoga maritima* | 2 |
|  | HAD_like | 1te2a | HAD-Like | *Escherichia coli O157:H7* | 1 |
|  | HAD_like superfamily | 1ta0a | HAD-Like | *Homo sapiens* | 3 |
|  | HAD_like | 3l5kA | HAD-Like | *Homo sapiens* | 3 |
|  | HAD_like | 3niwA | HAD-Like | *Bacteroides thetaiotaomicron* | 1 |
|  | HAD-like_dom | 1wpga | HAD-Like | *Oryctolagus cuniculus* | 18 |
|  | HAD-like_dom | 2amya | HAD-Like | *Homo sapiens* | 1 |
|  | HAD_like | 1swva | HAD-Like | *Bacillus cereus* | 3 |
|  | HAD_like | 2hsza | HAD-Like | *Haemophilus somnus 129PT* | 1 |
|  | HAD_like | 1nrwa | HAD-Like | *Bacillus subtilis* | 1 |
|  | HAD_like | 1ys9a | HAD-Like | *Streptococcus pyogenes M1 GAS* | 1 |
|  | HAD_like | 1qyia | HAD-Like | *Staphylococcus aureus subsp. aureus MW2* | 1 |
| Endonuclease/Exonuclease/phosphatase | EEP superfamily | 2j63B | Ap-Endonuclease | *Leishmania major* | 1 |
|  | EEP superfamily | 3tebA | Endonuclease/Exonuclease/Phosphatase | *Leptotrichia buccalis C-1013-b* | 6 |
|  | INPP5c | 3mtcA | Type II Inositol-1,4,5-Trisphosphate 5-Phosphatase | *Homo sapiens* | 6 |
|  | IMPase | 2hhma | Inositol Monophosphatase | *Homo sapiens* | 1 |
|  | Syja_N superfamily | 3lwtX | Phosphoinositide Phosphatase SAC1 | *Saccharomyces cerevisiae* | 2 |
|  | Metal Dependent Phosphatase | 1ka1a | Inositol Monophosphatase | *Saccharomyces cerevisiae* | 2 |
| Pyrophosphatase | PTPc | 3lqwA | Deoxyuridine 5'-Triphosphate Nucleotidohydrolase | *Entamoeba histolytica HM-1:IMSS* | 7 |
|  | trimeric_dUTPase |
|  | pyrophosphatase superfamily | 1e9ga | Inorganic Pyrophosphatase | *Saccharomyces cerevisiae* | 1 |
| Class I Cys-based PTPs | Y_phosphatase_m | 1zsqa | Phosphatase Domain | *Homo sapiens* | 6 |
|  | PTPc | 1D5RA | Phosphoinositide Phosphatase Pten | *Homo sapiens* | 6 |
|  |  | 3emuA | Leucine Rich Repeat And Phosphatase Domain Containing Protein | *Entamoeba histolytica HM-1:IMSS* | 1 |
|  |  | 1ywfa | Phosphotyrosine Protein Phosphatase Ptpb | *Mycobacterium tuberculosis* | 1 |
|  |  | 1v3aa | Protein Tyrosine Phosphatase Type IVA | *Homo sapiens* | 1 |
|  |  | 2jjdA | Receptor-Type Tyrosine-Protein Phosphatase | *Homo sapiens* | 1 |
|  |  | 1jlna | Protein Tyrosine Phosphatase | *Mus musculus* | 1 |
|  |  | 1fpza | Cyclin-Dependent Kinase Inhibitor 3 | *Homo sapiens* | 1 |
|  | DSPc | 1lw3A | Myotubularin-Related Protein2 | *Homo sapiens* | 10 |
|  |  | 2wgpA | Dual Specificity Protein Phosphatase 14 | *Homo sapiens* | 1 |
|  |  | 2oudA | Dual Specificity Protein Phosphatase 10 | *Homo sapiens* | 14 |
| Class II Cys-based PTPs | CDC25 (DSPc) | 2vswB | Dual Specificity Protein Phosphatase 16 | *Homo sapiens* | 4 |
|  |  | 1ymka | Rhodanese/Cell Cycle Control Phosphatase | *Homo sapiens* | 8 |
|  |  | 2gwfa | Rhodanase Domain | *Homo sapiens* | 2 |
| Class III Cys-based PTPs | LMWPTPASE | 3jviA | Protein Tyrosine Phosphatase | *Entamoeba histolytica HM-1:IMSS* | 2 |
| Histidine Phosphatase | HP superfamily | 2glcA | Histidine Acid Phosphatase | *Francisella tularensis subsp. holarctica* | 1 |
|  | HP superfamily | 2if8B | Inositol Polyphosphate Multikinase | *Saccharomyces cerevisiae* | 1 |
|  | HP superfamily | 3c7tB | HP Superfamily | *Bombyx mori* | 1 |
|  | PAP2_like | 1d2ta | Acid Phosphatase | *Escherichia blattae* | 11 |
|  | HP superfamily | 1h2ea | Histidine Acid Phosphatase | *Geobacillus stearothermophilus* | 1 |
|  | HP superfamily | 1nd6a | HP Superfamily | *Homo sapiens* | 3 |
|  | HP superfamily | 1qhfa | HP Superfamily | *Saccharomyces cerevisiae* | 1 |

**Table S4.** Classification of hypothetical proteins on the basis of InterPro domains with significant e-values.

| S.No. | Hypothetical Protein | Phosphatase Family | E-value |
| --- | --- | --- | --- |
|  | EAL51454.1 | PTPc superfamily | 7.0E-7 |
|  | EAL44282.2 | CDC25 (DSPc) | 9.2E-8 |
|  | EAL45023.2 | CDC25 (DSPc) | 3.4E-6 |
|  | EAL51514.2 | CDC25 (DSPc) | 0 |
|  | EAL43337.1 | CDC25 (DSPc) | 2.7E-4 |
|  | EAL46014.1 | CDC25 (DSPc) | 3.9E-5 |
|  | EAL45229.2 | CDC25 (DSPc) | 2.8E-12 |
|  | EAL48077.1 | CDC25 (DSPc) | 3.9E-8 |
|  | EDS89700.1 | CDC25 (DSPc) | 9.8E-7 |
|  | EAL48462.2 | PTPc superfamily | 2.6E-8 |
|  | EAL51319.1 | Dual specificity phosphatase, catalytic domain | 0.0019 |
|  | EAL47604.1 | Tyrosine phosphatase family | 1.0E-18 |
|  | EAL47515.2 | HP superfamily | 1.3E-28 |
|  | EAL50312.1 | HP superfamily | 1.3E-28 |
|  | EAL45556.1 | FCP1 homology domain | 2.7E-58 |
|  | EAL43056.1 | HAD_like | 3.4E-14 |
|  | EAL52001.1 | HAD_like | 1.5E-21 |
|  | EAL44593.1 | HAD_like | 1.9E-18 |
|  | EAL47582.1 | HAD_like | 5.1E-42 |
|  | EAL51181.2 | HAD_like | 2.5E-12 |
|  | EDS88666.1 | PPM Family (PP2C) | 1.9E-15 |
|  | EAL50496.2 | PPM Family (PP2C) | 2.6E-11 |
|  | EAL46019.1 | PPM Family (PP2C) | 1.2E-11 |
|  | EAL47483.2 | PPM Family (PP2C) | 3.6E-6 |
|  | EAL51376.1 | PPM Family (PP2C) | 7.1E-12 |
|  | EAL46566.1 | PPM Family (PP2C) | 2.3E-10 |
|  | EAL47323.1 | PPM Family (PP2C) | 6.6E-9 |
|  | EAL44998.1 | PPM Family (PP2C) | 1.0E-12 |
|  | EAL43601.2 | PPM Family (PP2C) | 6.7E-10 |
|  | EAL47829.1 | PPM Family (PP2C) | 1.3E-6 |
|  | EAL47810.1 | PPM Family (PP2C) | 8.5E-12 |
|  | EAL43164.1 | PPM Family (PP2C) | 2.2E-5 |
|  | EAL49020.1 | PPP Family (PP2A) | 0.05 |
|  | EAL46647.2 | PPP Family | 1.5E-13 |
|  | EAL45044.1 | PPP Family | 1.6E-4 |
|  | EAL46766.1 | EEP superfamily | 1.1E-5 |
|  | EAL46465.1 | EEP superfamily (Syja_N) | 0 |
|  | EDS88629.1 | DeoxyUTP pyrophosphatase Family | 7.2E-51 |
|  | EDS88738.1 | DeoxyUTP pyrophosphatase Family | 3.9E-49 |
